# Using consumer sleep trackers for sleep extension interventions: retention, adherence, and user experiences

**DOI:** 10.1093/sleepadvances/zpag025

**Published:** 2026-02-16

**Authors:** Eliza Taylor, Mary Takgbajouah, Jennifer Duffecy, Minsun Park, Sirimon Reutrakul, Pamela Martyn-Nemeth, Kelly Glazer Baron

**Affiliations:** Department of Family and Preventive Medicine, University of Utah, Salt Lake City, UT, United States; Department of Biobehavioral Nursing Science, College of Nursing, University of Illinois, Chicago, IL, United States; Department of Psychiatry, University of Illinois, Chicago, IL, United States; Department of Biobehavioral Nursing Science, College of Nursing, University of Illinois, Chicago, IL, United States; Department of Medicine, University of Illinois, Chicago, IL, United States; Department of Biobehavioral Nursing Science, College of Nursing, University of Illinois, Chicago, IL, United States; Department of Family and Preventive Medicine, University of Utah, Salt Lake City, UT, United States

**Keywords:** wearables, sleep, technology

## Abstract

**Study Objectives:**

Consumer sleep trackers (CSTs) can provide insights into sleep behaviors for behavioral interventions. This study aimed to describe the implementation, adherence, and participant perceptions of CSTs across two behavioral sleep extension studies: Study 1, the Sleep Technology Intervention to Target Cardiometabolic Health and Study 2, the Sleep Optimization for Type 1 Diabetes.

**Methods:**

Both studies employed randomized controlled trial designs. Participants were provided with CSTs and engaged in structured coaching sessions via Zoom or phone, supplemented with sleep education materials. Adherence was measured by the number of days with sleep data recorded during the intervention periods (8 weeks for Study 1, 12 weeks for Study 2). Participant feedback was collected through end-of-study surveys that included open-ended questions with free text answers to assess usability, satisfaction, and perceived impact of CSTs. Open-ended feedback was analyzed using reflexive thematic analysis.

**Results:**

Study 1 enrolled 60 participants, and Study 2 enrolled 73 participants. Adherence was 89% and 86% and retention was 100% and 86%, respectively. Open-ended feedback revealed three themes: positive aspects of device use, negative aspects of device use, and device usability and data interactions. Participants found the CST helpful for motivation and goal tracking. However, technical issues, discomfort, and emotional responses to sleep data were noted as barriers.

**Conclusions:**

CSTs, when paired with personalized coaching and support, can effectively be used to promote participant engagement in sleep behavior change studies. Continued research is essential to refine wearable technology interventions to maximize their impact on health behavior modification.

This article is part of the Consumer Sleep Technology Special Collection

**Statement of Significance**

Consumer sleep trackers have potential to increase sleep awareness and facilitate sleep behavior change. By examining their implementation, adherence, and user perceptions, we can enhance their effectiveness and optimize their integration into sleep health interventions.

## Introduction

The use of consumer sleep trackers (CSTs) has rapidly increased in the population, particularly in the United States. Data from the past 5 years suggests that one in three American adults owns a fitness tracker, with most having the capacity to estimate sleep.^1^ Apple watches (44%) and Fitbits (21%) are the most commonly used devices in recent studies.^2^ Although not all individuals purchase devices to track sleep, sleep is commonly rated as one of the most desired features of wearable devices.^3^ In our previous survey of individuals with short sleep duration, CSTs were a highly desired method of increasing sleep duration, compared to meeting with a coach, and were favored by 84% of the sample.^4^ A recent study of device users demonstrated that younger, female, and higher-income participants were more likely to use devices and 81% favored sharing the data with their physician,^5^ which demonstrates the potential for use of this data in clinical interventions and potential imbalances in who is seeking this tool.

The growing popularity of CSTs demonstrates their potential to engage patients in interventions in creative ways.^6^^,^^7^ Leveraging the population interest in tracking sleep, Drs. Baron and Duffecy have been developing and testing a behavioral sleep intervention using CSTs, brief telephone coaching, and educational material.^8^ Over the past decade, our team has conducted development and pilot studies to refine our intervention and test CSTs in different populations who may benefit from extending and improving their sleep, including individuals with hypertension, a history of gestational diabetes, and type 1 diabetes.^9–11^ These studies have provided opportunity to refine our intervention and develop valuable experience in different populations using CSTs in interventions as we have moved toward larger randomized studies of our intervention.

The purpose of this article is to describe the implementation of CSTs in behavioral sleep interventions and evaluate adherence, feasibility, and perceptions of CSTs in two randomized controlled trials: the Sleep Technology Intervention to Target Cardiometabolic Health (STITCH) and the Sleep Optimization for Type 1 Diabetes (Sleep-Opt) studies.^12^^,^^13^ The STITCH study is a single-blind trial designed to assess the effects of a behavioral sleep extension intervention on sleep duration and cardiometabolic health among adults with habitual short sleep duration (<7 h per night) and elevated blood pressure (BP) (SBP 120–150 mm Hg or DBP 80–90 mm Hg).^1^ The Sleep-Opt study is a single-blind trial aimed at reducing sleep variability and insufficient sleep duration, with the goal of improving glycemic control in working-age adults with type 1 diabetes (T1D) and short sleep duration or irregular sleep timing. Both studies utilized CSTs, brief telephone coaching, and education to track progress and set goals for participant’s sleep. These two trials have recently concluded and are currently being analyzed. This paper explores the role of CSTs in behavioral sleep extension interventions, highlighting advantages and barriers while centering qualitative feedback to better understand their practical implications. Both wear time and subjective experience reported via free text responses are important to understanding feasibility and acceptability as well as factors that promote CST engagement in research. As CSTs are becoming more common among sleep research, both observational and interventions, promoting adherence to device use and improving user experience is important to collecting high quality data and delivering interventions utilizing CST data.

## Methods

### Participants and procedure

#### Study 1: STITCH study

The University of Utah Institutional Review Board (IRB_00130332) approved this research. Full details of the methods are reported here.^12^ Briefly, individuals in Salt Lake City and the surrounding area were invited to participate in the study from the University of Utah Health (UU Health) primary care, paid online advertisements, community clinic posters, and at community health fairs. Potential participants completed online eligibility prescreening and if potentially eligible, completed in lab BP measurement, 24-h ambulatory BP, 7 days of actigraphy to verify sleep duration criteria, and one night of an at home sleep apnea screening. Inclusion criteria included the following: aged between 18 and 65 years; elevated in-office BP readings (systolic BP of 120–150 mm Hg or diastolic BP of 80–90 mm Hg); time in bed less than 8 h with a sleep duration of less than 7 h; regular smartphone use; and the ability to read and write in English. Participants were excluded if they had other moderate to severe comorbid sleep disorders (e.g. obstructive sleep apnea, restless legs syndrome, or insomnia); resistant hypertension, defined as taking more than four antihypertensive medications or three medications with standardized in-lab BP greater than 130 mm Hg systolic or 80 mm Hg diastolic at screening; unstable or serious medical or psychiatric illnesses that could interfere with participation (e.g. cancer, renal disease requiring dialysis); overnight work more than once per month; use of hypnotic or stimulant medications; or situations that would significantly impact the ability to extend sleep, including pregnancy, overnight caregiving responsibilities for children or adults more than once per week.

#### Study 2: Sleep opt study

This University of Illinois Institutional Review Board (IRB Protocol 2020-0374) approved this research. Individuals across the U.S were invited to participate in the study through the University of Illinois at Chicago Medical Center, local diabetes clinics, diabetes-related websites and organizations, and social media. Recruitment methods included flyers, e-announcements, recruitment letters, listservs, Research Match, outreach to participants from previous studies who had consented to future contact, and collaboration with the Division of Endocrinology at Cook County Health. All interested individuals were screened for eligibility and were required to meet the following criteria: aged between 18 and 65 years; clinical diagnosis of T1D for at least 1 year; and reported habitual sleep variability (>1 h variation in sleep onset or wake up time/week) or insufficient sleep duration (<6.5 h/night during work- or weekdays). Participants were excluded if they scored ≥15 on the Insomnia Severity Index; were at high risk for obstructive sleep apnea; had a history of severe hypoglycemia; had an glycated hemoglobin (HbAIC) > 10%; were rotating shift or night shift workers; used sleep medications or aids; had significant renal impairment or significant medical morbidities; or were breast feeding, pregnant, or planning pregnancy.

### Intervention procedure

Although both studies employed Fitbits, however the interventions were designed to be delivered based on any user generated sleep data, either device or sleep diary. The following CST setup procedure was implemented in both studies. Participants randomized to the intervention group received an email and phone call from the study coach, along with a mailed packet containing a letter, CST, and setup instructions (Appendix A). To ensure data deidentification, the research team created study-specific email accounts for each participant. The setup instructions included step-by-step guidance on downloading the Fitbit app, logging in, and wearing, charging, and syncing the device, supported by screenshots and descriptive text. Additionally, participants received information on how to navigate the app dashboard to view sleep data and manually edit sleep logs. During the initial coaching call, coaches reviewed the setup instructions as needed and confirmed participants’ understanding. The coach and/or project coordinator connected each device to the Fitabase platform (Google, Mountain View, CA) to track and aggregate participants’ data. Within 1 week of device distribution, the coach contacted each participant to verify that the Fitbit was properly syncing with both the mobile application and the Fitabase platform. If participant data were not visible to the coach, a troubleshooting protocol was followed via phone beginning with verification that the app was installed and login credentials were correctly entered. Subsequently, a syncing checklist was implemented, which included the following steps: (1) confirming that Bluetooth was enabled on the participant’s mobile device, (2) opening the Fitbit app, and (3) manually selecting “Sync Now” in the top right-hand corner.

Participants met with coaches weekly during the intervention period either via zoom or telephone. The first session lasted approximately 20 min to introduce the program and establish reasons for change and program goals, and subsequent sessions lasted 5 min. During each meeting, the coach reviewed the participant’s sleep data from the previous week using the Fitabase dashboard. The coach and participant discussed sleep-related challenges and facilitators and collaboratively set a sleep goal for the upcoming week. These goals typically included establishing a new bedtime or wake time, reducing procrastination behaviors, enhancing the sleep environment, and developing consistent and healthy bedtime routines. The coaching protocol was based on the principles of Supportive Accountability and used techniques drawn from cognitive behavioral therapy and motivational interviewing, such as goal setting and identifying discrepancies between current and desired behaviors.^14^^,^^15^ The health coach was trained to set collaborative goals, elicit change talk, and include relevant sleep-related research in their sessions (e.g. even an improvement of 20 min can improve your performance). In addition to the coaching calls, participants also received sleep lessons via email on the same cadence as the calls. Article topics included effects of blue light, sleep procrastination, and long-term health benefits of sleep. The difference in study duration was related to the study outcome. Sleep related changes in BP have been demonstrated for shorter intervention periods,^9^ whereas changes in glycated hemoglobin, the Sleep-Opt study’s main outcome measure, a standard clinical parameter of glycemic control in the preceding 90 days, requires 3 months to demonstrate change.

### Measures

Intervention Retention was determined by number of coaching sessions attended. The study goal was to attend 80% of sessions. Missed sessions were rescheduled at the next available time. CST adherence was defined as the frequency of days available via the participant’s Fitbit.com account in the intervention period. Any day with non-missing Fitbit data was considered a valid day.

#### Participant feedback

At the 6-month follow-up visit, participants completed a series of open-ended questions through an electronic REDCap survey. Participants were provided with questions and a free-text option to respond. Responses were not required for these items. STITCH participants were asked: (1) What would make you more likely to look at your sleep data at least daily?; (2) Did Fitbit help you make any insights about your sleep?; (3) Were there any ways Fitbit was not helpful or interfered with your sleep?; and (4) Do you have any specific feedback about your experience of using the Fitbit?

Sleep Opt participants were asked at the end of the 12-month study: (1) How did you find the content of the study and its purpose?; (2) What did you think of the style/organization of the study?; (3) What did you think of the coaching program? Was it helpful? Did you use any of the suggestions in your everyday life?; (4) What did you enjoy most about the study?; (5) What do you think could be improved about the study or its delivery?

#### Analysis

We analyzed CST use patterns via descriptive analyses. Patterns of Fitbit CST usage were examined using descriptive statistical analyses to summarize engagement and usage characteristics. Qualitative data from open-ended questions from both studies were analyzed using reflexive thematic analysis (RTA), following Braun and Clarke’s approach.^16^^,^^17^ RTA allows for researcher reflexivity and iterative theme development rather than rigid coding reliability. All responses from STITCH and CST-related responses from Sleep Opt were included.

Initially, ET, MT, and KB first engaged in repeated reading of the qualitative feedback and collaboratively developed a preliminary, flexible set of early codes based on emergent concepts from the data; these codes were revised throughout analysis, consistent with RTA’s non-codebook orientation. ET and MT independently coded the full dataset using this framework, generating new codes and modifying existing codes when needed. Subsequently, all codes were reviewed, refined, and discussed and approved by KB and full research team to ensure consistency and depth of interpretation. Discrepancies or differences in interpretation were used productively and were collaboratively constructed throughout the course of analysis. Final themes were iteratively developed through ongoing reflexive dialogue to capture patterns across responses.

## Results

Intervention adherence in both studies was high, indicating that most participants attended the coaching sessions (Table 1). Participants were also highly adherent to wearing the CSTs, and coaches successfully connected to the devices for all intervention participants. With clear instructions and personalized support, participants in both studies were able to set up and sync their devices with minimal data loss.

**Table 1:** Participant characteristics and CST usage

Sample characteristics	STITCH (*n* = 60), 8 weeks M (SD) or *N* (%)	Sleep-Opt (*n* = 73), 12 weeks M (SD) or *N* (%)
Age	42.2 (11.3)	38 (11.8)
Sex Male Female	35 (58%) 25 (42%)	24 (33%) 49 (67%)
Race American Indian/Alaska Native Asian Black or African American Native Hawaiian or Pacific Islander White More than one race Did not wish to disclose	1 (2%) 7 (12%) 4 (7%) 0 (0%) 43 (72%) 4 (7%) 1 (2%)	— 3 (4%) 9 (12%) — 57 (78%) 4 (6%) —
Ethnicity Not Hispanic or Latino Hispanic or Latino	52 (87%) 8 (13%)	67 (92%) 6 (8%)
Education Did not graduate high school High school graduate and/or Some college Bachelors (4 year degree) Graduate degree or more	— 17 (28%) 16 (27%) 27 (45%)	1 (1%) 11 (15%) 35 (48%) 26 (36%)
Intervention adherence	59 (100%)	63 (86%)
Days watch worn	56 (89%)	62 (74%)

In the STITCH study, Participants reported enjoying the CST and looked at the data often (Table 2). However, only 24% reported looking at it daily in the past 6 months. Overall, participants in the intervention group reported being satisfied with their study group (average 81 out of 100). This implementation of this survey was incorporated after the study began so not all participants received the survey, therefore responses were only available for 54 participants.

**Table 2:** Intervention ratings in the STITCH Study (*N* = 54)^*^

Question		*N* (%) or M (SD)
How much did you enjoy wearing the Fitbit these last 6 months?	Never Rarely Sometimes Frequently Always	2 (7%) 0 (0%) 6 (21%) 13 (45%) 8 (28%)
In the past 6 months, how often did you look at your sleep data on the Fitbit app?	Never 1–2 days per week More days than not Daily More than once a day	0 (0%) 6 (21%) 16 (55%) 7 (24%) 0 (0%)
How satisfied were you with your study group? (rated from 1 to 100)		81.5 (15.7%)

^*^Missing data *n* = 25 due to adding the questionnaire after data collection had begun.

### Participant feedback

Participant feedback was often brief, which constrained the depth of the themes that could be developed. Because participant perspectives were highly consistent across the two studies, the following derived themes are presented jointly: positive aspects of device use, negative aspects of device use, and device usability and data interactions (see Supplementary Table S1).

#### Positive aspects of device use

Responses indicating ease of use, insightfulness, comfort, or helpfulness in improving sleep patterns were categorized as Positive feedback about CST use. Participants from both studies reported the CST data to be useful in developing insight into their insufficient sleep duration. Many expressed that it was helpful to learn that time in bed does not equate to hours asleep (e.g. 8 h in bed is not 8 h of sleep). Several participants found the sleep metrics helpful for showing improvement after changing their sleep patterns.

“I think that the most interesting bit is how after changing my sleep environment how much better my sleep was.”—STITCH Study Participant

“Yes, it [CST] helped me know when I fall asleep each night and what times I wake up in the middle of the night. For example, I seemed to wake up less when I went to bed earlier rather than later.”—STITCH Study Participant

Participants also reported that wearing the CST and reviewing their sleep data was motivating, and they appreciated the device’s comfort and ease of use, particularly its ability to sync data directly to their sleep coach, discuss the data and develop greater awareness about their sleep patterns.

“I enjoyed watching my sleep quality progress. Also, I felt like through the study I became less pessimistic about my sleep, which helped me sleep better overall.”—Sleep Opt Participant

#### Negative aspects of device use

Responses describing examples of frustration, discomfort, perceived data inaccuracies, or intrusiveness were categorized as Negative Feedback. Some participants reported technical difficulties, including challenges with data syncing and remembering to charge the device or questioned the accuracy of the sleep tracking, noting instances where the device appeared overly sensitive.

“Battery recharges have been hard to work out. There have been a few times I’ve struggled to have it charged in time for bed.”—STITCH Study Participant

“Not helpful—too sensitive, says I’m awake when I am probably just turning over. It constantly says I was awake when I didn’t think I was.”—STITCH Study Participant

In terms of usability, three participants found the device uncomfortable to wear during sleep, with one requesting a fabric replacement band. Device durability was another concern; five CSTs in the STITCH study were reported as broken or lost and required replacement. Some participants also noted feeling discouraged when viewing their sleep data independently, citing limited time, lack of motivation, unclear perceived value of the metrics, and emotional discomfort from observing unmet sleep goals. Only one response discussed the duration of wearing the CST and suggested that 12 weeks was too long.

“Wearing a Fitbit to bed everyday for 12 weeks is too long. The goal should be to wear it for 4-5 days a week and not a constant 7 days. Sleeping with something makes it a less pleasant sleep.”—Sleep Opt Participant

#### Device usability and data interactions

Responses related to the device itself or to navigating the data were categorized under device usability and data interactions. These comments stemmed from questions about how to improve insight into the data, potential enhancements to the device, the usefulness of external reminders or scheduling, and the influence of incentives or curiosity on data engagement. The STITCH study generated a greater volume of feedback regarding how often participants engaged with their data, suggestions for enhancing the insights provided by the device data, and recommendations for increasing participants’ engagement with the data, because participants were specifically asked questions in these domains. Participants provided feedback on what features gave them the most insights or features they would like to see (e.g. one participant would have liked to see real time heart rate or oxygen saturation). They also commented on the need to block out time or have another reminder to view their data (STITCH study only). Participants in both studies commented that seeing how much they slept was beneficial and motivating.

“A notification in the morning or a banner across the screen with a quick view of sleep data [would prompt me to check it more often].”—STITCH Participant

### Coaching feedback

Responses from the Sleep Opt study were more likely to discuss coaching. Most responses were positive and discussed the helpfulness of discussing their data with a coach.

“It was very insightful on how my sleep improved over the 12 weeks with the coaching sessions.”—Sleep Opt Participant

However, one participant reported the coaching sessions were not helpful and would have preferred a more structured curriculum.

“Not as helpful as I had hoped, the check ins with the sleep coach weren’t very useful and felt more like brief chats then any sort of sleep class.”—Sleep Opt Participant

### Coaching strategies

To enhance CST usage among participants, our team implemented several strategies:

#### Accessible support

Weekly calls with the health coach provided a frequent and structured opportunity for accountability, support, and technological trouble shooting with the CSTs. By emphasizing not only areas in need of improvement but also highlighting positive sleep trends, reinforced progress and encouraged continued adherence to wearing the device. Coaches were readily available to participants via email and phone to answer any questions that came up between coaching sessions. Participants reached out between sessions to reschedule coaching calls, notify of periods they would be unable to wear the watch, or ask miscellaneous sleep questions often prompted by the sleep education materials such as “Do you have mattress recommendations”. While participants reached out somewhat regularly to reschedule, other support requests were infrequent.

#### Responsive and creative feedback

Coaches worked with participants to quickly troubleshoot any issues that came up. For example, when a participant lost their CST, the coach sent a new one. In cases of watch band discomfort, the team purchased a fabric band and shipped it directly to the participant. If a participant could not remember to charge their watch, coaches worked to develop charging schedules with phone alert reminders.

#### Data feedback and goal setting

Finally, goal setting was a key strategy utilized to encourage watch wearing. By setting a new goal every week, participants had a constant reason to be wearing the CSTs and interacting with the data. Many participants reported increased motivation to improve their sleep because they knew their data was being reviewed and analyzed by the coach. On weeks where participants missed days of wear, the coach spent time discussing the challenges that occurred that prohibited watch wearing and reemphasized the importance of watch wearing to properly assess the personalized goals.

### Barriers

Despite these supportive efforts, several barriers affected sustained device usage. If the participant was not motivated to improve their sleep, coaches found strategies such as data feedback and goal setting unsuccessful in enhancing sleep and watch adherence. Technical complications, including syncing issues with Fitabase or the Fitbit mobile platform, as well as perceived inaccuracies in sleep tracking, diminished engagement. Physical discomfort while wearing the device overnight also discouraged adherence. Coaches encountered difficulties in fully addressing these obstacles, particularly when data was unavailable due to communication errors between participants’ Fitbit accounts and the Fitabase system. In some cases, these issues limited the ability to provide timely and tailored feedback during coaching sessions.

The use of the Fitabase platform was easy for our coaches to set up and follow-participants data but there are limitations in the granularity of data that can be downloaded. Finally, due to Fitbit’s new integration with Google, creating and managing research-specific participant accounts is now more complex and requires additional steps.

## Discussion

### Implications for wearable technology use

The integration of CSTs into behavioral sleep extension interventions, as demonstrated in the STITCH and Sleep Opt studies, highlights both the potential and the challenges of using wearable activity trackers to support health behavior change in future research studies. Results of our study demonstrate high adherence to wearing CSTs in two recent randomized studies of coached interventions utilizing the CST data to motivate behavior change. With clear instructions and personalized support, participants in both studies were able to set up and sync their devices with minimal data loss. Given the tendency for CST users to be more predominantly female, our study demonstrated high use in both genders, without noted sex differences. These findings underscore the importance of addressing both behavioral and technical challenges to optimize wearable device interventions. Consistent with our previous study, most participants responded favorably to utilizing a CST in the intervention. Despite having somewhat different open-ended questions, the two studies had mostly consistent feedback. In our previous research, the CST was often viewed as the most desirable aspect of our intervention by some participants.^18^ However, technical challenges were present for some participants, as was discouragement. It is also important to note that none of the participants reported increased anxiety with tracking their sleep, or orthosomnia, because of wearing the tracker.^19^ Negative feedback tended to be focused on discomfort or inconvenience of remembering to charge it. It is notable that the only comment about duration of wear time was in the Sleep Opt study, which was 12 weeks compared to the 8-week STITCH study, however this was only one participants’ opinion.

Few studies have evaluated CST wear time in sleep intervention studies, in part because using CSTs in sleep interventions is relatively uncommon beyond our groups research. In a previous factorial study from our group, we examined the effects of the CST, CST plus coaching, coaching alone and self-management. The CST group wore the device 88.2% of days and the Fitbit + Coaching group wore the device 93.9% of days, which suggests a benefit of the coaching for CST adherence.^18^ Far more studies have published wearable tracker adherence in physical activity research. A recent systematic review of studies using Fitbit devices demonstrated adherence from 60% to 97% of days among studies ranging from 7 days to 48 weeks.^20^ Techniques to promote adherence included phone calls and providing summaries of weekly and daily physical activity were commonly used to promote device wear.

It is important to consider that our interventions involved weekly interaction with a coach, who was able to troubleshoot any issues that presented with the device and utilized the device data to motivate behavior change. Future research should examine design elements of delivering sleep extension to improve scalability, such as interventions including understanding the number of sessions, whether coaching should be synchronous vs asynchronous and testing the use of automated coaching such as chatbots. A meta-analysis of internet-based psychotherapy interventions suggested that therapist contact improved outcomes.^21^ However, coaching did not improve results of a physical activity promotion app.^22^ Overall, health behavior change research has an opportunity to understand how to blend technology-assisted health behavior change interventions with human interaction, to determine which intervention is effective based on population, behavior, and context.^22^

### Limitations

The frequency of coaching is a strength and limitation of our study. Not every study has the staffing for this degree of 1:1 support, thus, our findings may not be generalizable to research interventions that do not incorporate a health coach or CST use in daily life, outside of an intervention. Additionally, our study sample may limit generalizability to populations with lower education or limited smartphone access. In other currently ongoing studies in our lab that enroll in a wider range of incomes (1R01MD018517, R33 AG084477), we utilize study phones that can be loaned to participants to complete the protocol and using the procedures described in this study, we have been successful at supporting participants of a range of ages, educational and technology abilities to utilize CSTs. Finally, the qualitative responses in our study were in response to short-answer, written questions, and using focus groups or interviews may have produced more detailed information and illustrative quotes about facilitators and barriers to CST use.

## Conclusion

Results of this study demonstrate feasibility and problem solving to utilize CSTs to deliver sleep extension interventions among two chronic illness populations. As wearable technology evolves, further research on CSTs usage and adherence is essential to enhance their effectiveness in promoting healthy behaviors and improving overall well-being.

## Supplementary Material

Supp1_qualitative_themes_updated_R2clean_zpag025

## Data Availability

Data are available upon request to the corresponding author.
