# Supplementary material for: Using consumer sleep trackers for sleep extension interventions: retention, adherence, and user experiences
Source: Sleep Adv. 2026 Feb 16;7(2):zpag025. doi: 10.1093/sleepadvances/zpag025 (PMC13116327; doi:10.1093/sleepadvances/zpag025)
Supplement: Supp1_qualitative_themes_updated_R2clean_zpag025 [file supp1_qualitative_themes_updated_r2clean_zpag025.docx]

Using Consumer Sleep Trackers for Sleep Extension Interventions: Retention, Adherence, and User Experiences

Eliza Taylor^1^; Mary Takgbajouah^2^, Jennifer Duffecy^3^; Minsun Park^2^, Sirimon Reutrakul^4^; Pamela Martyn-Nemeth^2^; Kelly Glazer Baron, PhD^1^

1.Department of Family and Preventive Medicine, University of Utah, SLC, USA

2. Department of Biobehavioral Nursing Science, College of Nursing, University of Illinois, Chicago, USA

3. Department of Psychiatry, University of Illinois, Chicago, USA

4. Department of Medicine, University of Illinois, Chicago, USA

Corresponding Author:

Kelly Glazer Baron, PhD, MPH, DBSM

310 Wakara Way, Suite 1100

Salt Lake City UT 84108

Kelly.baron@utah.edu

**Supplementary Table 1. Qualitative Responses**

| **Theme** | **Sub-Theme** | **STITCH Quotes** | **Sleep Opt quotes** |
| --- | --- | --- | --- |
| **Positive- insightful or helpful** | Insights | - Yes (x5 responses) - That a lot of my sleep is restless. - Yes, 8 hours of sleep does not equal 8 hours of actual sleep. - Yes, It helped me to see how much effective sleep I was getting. - How often I wake up during the night. - Yes, I could how long it took to fall asleep, or how many times I woke up at night. - It helped to keep me accountable - Yes! I knew how good my sleep actually was - Only that I am a pretty deep sleeper. - Yes. having a routine matters very much. my quality of sleep is so much better when I stick to it! - It showed how much more often I woke up than I thought. - Yes, it gave me a lot of ideas on how my sleep was going and what was and wasn’t helping - Yes.  I got to see how much I slept versus awake time. - Yes, I didn't realize how much awake time occurred when I thought I was asleep. really shows how little sleep I must have been getting. - Yes it did. now I look forward to sleep early. it reminds me - Yes it made me realize how I am not getting enough sleep - Yes, it helped me know when I fall asleep each night and what times I wake up in the middle of the night. For example, I seemed to wake up less when I went to bed earlier rather than later. - I think that the most interesting bit is how after changing my sleep environment how much better my sleep was. - Yes, that according to it I am not getting as much sleep as I think I am - I didn't realize how little sleep I was actually getting. | - That it helped with my sleep routine - It was good. Sleep coach was helpful to process and integrate information. - I enjoyed the data that I got from the Fitbit (when it worked). - Tracking my sleep patterns - Trying out the fitbit sleep tracking technology. - Seeing the Fitbit data about how my sleep looks every night was interesting.  I also like thinking that maybe the data gathered might help other people - wearing the fitbit. |
|  | Helpful | - It is helpful to see the sleep patterns. It was pretty easy to set up and get used to. I love being able to look at my sleep quality and rhythms. Although it occasionally registers my 'toilet time' during the night as sleep. Guess I am very relaxed... - It's nice to be able to track my sleep. - None, I like using it to tell the quality of sleep. - It's very easy to use and i see everything on the fitbit - The fitbit has helped me track my sleep much better than I was before. I can see when I tend to wake up during the night. I feel that I have woken up less often while using the fitbit. - Me ayuda a encorvarme en la meta del horario para dormir. Loose translation: it helps me work toward the goal of my sleep schedule. - I found myself doing more of the healthy habits than just sleep related ones. - It's been great! It's really helped me established a pre-bedtime routine. The stats are fun to look at and help me understand my sleep better. | - Learning about how important a good sleep is. And using the Fitbit - Getting sleep data and have me a greater awareness of my sleep habits. - I enjoyed watching my sleep quality progress. Also, I felt like through the study I became less pessimistic about my sleep, which helped me sleep better overall. |
| **Negative-interfered or not helpful** | No insights or did not interfere | - No (12 participants) - No, being able to adjust the night time settings was great and very helpful and being able to check the time without having to turn was helpful too. - Nope. it was small and unobtrusive. - It did not interfere with my sleep.  But as mentioned above, sometimes the information wasn't accurate. - Not really - Not beyond what it was doing with my personal fitbit before I joined the study. - No.  Used it more for my daily activity.  There were times when the Fitbit didn't correctly record when I went to bed or woke up so I didn't rely on the sleep data because I wasn't sure if it was correct. - The Fitbit didn't really help me make any insights. | - Not as helpful as I had hoped, the check ins with the sleep coach weren't very useful and felt more like brief chats then any sort of sleep class. |
|  | Uncomfortable | - The band was not comfortable. - I didn't love it on my wrist to fall asleep, but it was okay to get used to - I had to adjust my position in order to not disturb my wife if the face of the Fitbit lit up - Yes, its uncomfortable - It took me a while to get used to wearing the Fitbit at night but after I did, I barely noticed it - It simply was uncomfortable to sleep with. | - The band was uncomfortable compared to other wrist-worn devices. - Wearing the Fitbit made it uncomfortable to sleep. - It needs to be made easier for the individual. Wearing a Fitbit to bed everyday for 12 weeks is too long. The goal should be to wear it for 4-5 days a week and not a constant 7 days. Sleeping with something makes it a less pleasant sleep. - It was fine but not a huge fan of wearing the watch to sleep. It was very uncomfortable |
|  | Inaccurate | - Not helpful - too sensitive, says I'm awake when I am probably just turning over. It constantly says I was awake when I didn't think I was - Didn’t give me enough information - Not really but kind of a difficult to use and adjust data when travelling - Didn't interfere, but not always accurate- it would say the wrong hours for sleep time when I clearly knew it was incorrect | - I would have really enjoy the Fitbit and tracking my sleep and exercise but the Fitbit did not work properly. :( |
|  | Charging Challenges | - Battery recharges have been hard to work out. There have been a few times I've struggled to have it charged in time for bed. - It was sometimes tricky to find a good time to charge it, out remember to put it back on before bed - I didn’t have any issues using the Fitbit aside from remembering to charge it.  As I was using the step tracking, and food tracking it was hard to find a time to let it charge up. |  |
|  | Poor Device/  Challenge | - A few times it seemed to not track my sleep very well, but I think we are beyond that now, and it seems to be doing better. Fitbit isn't a great device. Would recommend a better tool with less problems.  (response from general feedback) - More specific ways to fall back asleep when you wake up in the middle of the night. - I normally don't wear a watch, so it has been a slight challenge to get use to wearing....especially while sleeping. - Devise is poor quality and doesn't seem to record information correctly at times. | - Use of fitbit data. My fitbit account was somehow not able to be linked or to view sleep data, so that did not end up being a helpful part of my participation in the study. - Fitbit for 12 weeks is a long time to wear to bedside especially if you're use to wearing nothing on your wrist while sleeping. |
| **Device usability and data interactions** | Ideas for enhanced insights | - It would be cool to have access to things like heart rate and Sp02 levels throughout the night. - The metrics and seeing how well I actually slept (current feature) - Need to track using a database other than the Fitbit tracker [would provide more insight]. - If there was a clearer explanation behind the sleep score [it would improve my insights] - A notification in the morning or a banner across the screen with a quick view of sleep data [would prompt me to check it more often]. - If it could be seen through my phone [I would gain more insights]. | - I enjoyed reviewing the data and my progress with my coach. - It was very insightful on how my sleep improved over the 12 weeks with the coaching sessions. |
|  | Device Improvements | - Having a better device [would improve my experience].  Fitbit is an average quality device. Sleep data was inaccurate at times. - If the fitbit was more comfortable to wear at night - Not having to resync several times before viewing the data. - If I had access to the pro account. | - The sleep info on the FitBit app was good, not great |
|  | Need for external reminder/schedule | - If I had a reminder on my Fitbit [I would check it more often]. - A blocked out time to review it [I would check it more often]. - If i had to track it for some reason [I would check it more often] - Someone asking for details about my sleep, or something that forces me to retrieve sleep data information. - I think having a set time to examine my data would help me look at my sleep data daily. - I look at it daily. - for the first several months I did, then I was able to gage my sleep quality a little bit better, even without checking. - Not sure, I'm sick of notifications on my phone. - There really wasn't any specific thing that made me check. - Already do that [check my data daily]. |  |
|  | Incentives and intrinsic curiosity | - Curiosity [reason for checking data] - Give me money each time I looked [would make me check it more often]. - So I can make sure I get enough sleep [is why I check my data]. - Curious to see how long I slept and to see if I met my goal - I checked the hours on occasion, but focused more on other aspects of the fitbit to get my life in better shape overall. - An actual reward for looking at the data (not virtual reward), - How tired I was during the day [would motivate me to check my data]. | - Wearing the Fitbit was one of the best parts of the study. - Getting sleep data and having greater awareness of my sleep habits was very beneficial. - Actually seeing and quantifying how much sleep I was getting [was helpful] - Seeing my sleep habits [was helpful] - The fitbit [was the best part of the intervention]. It motivated me to improve. |

Note. Responses above are based on open ended questions presented in each study.
